# Chronic Gonorrhœa in the Male and Its Treatment

**Published:** 1893-06

**Authors:** Floyd W. McRae

**Affiliations:** Atlanta, Ga.


### CHRONIC GONORRHŒA IN THE MALE AND ITS TREATMENT.*

By FLOYD W. McRAE, M. D.,
ATLANTA, GA.

MR. PRESIDENT AND GENTLEMEN: In the short notice given me I have found it impossible to prepare a paper for the evening but have consented to open the discussion.

*Delivered before the Atlanta Society of Medicine. Stenographically reported.

The subject I have is "Chronic Gonorrhœa in the Male and its Treatment." I hope that the discussion will include not only the treatment of chronic gonorrhœa in the male but also its bad effects in the female. That is one of the main reasons we should cure it—the ultimate bad effects it produces in the female. The universal prevalence of this disease, its intractability to treatment, the direful results following in its wake when unrecognized, or unsuccessfully treated, are the reasons which have prompted me to bring this subject before you for discussion. Just so long as nature is determined that the race shall be propagated at all hazards, just so long will the passions of men make them disregard not only the teachings of moralists but also the health and even the lives of others. Notwithstanding these facts, much can be done by the conscientious physician to impress upon the victim of this terrible malady the moral turpitude of the man who would willingly and knowingly communicate it in all its loathsomeness to an unsuspecting female, whether she be his wife or his mistress.

It cannot be denied that a very large proportion of the ills resulting directly or indirectly from chronic gonorrhœa are chargeable to either the ignorance or carelessness of physicians. They are too prone to tell the victim of acute gonorrhœa, who expects to be cured in a few weeks at longest, that he is well as soon as the blenorrhœa has ceased and to lead him to believe that the recurrences from sexual or alcoholic intemperance are reinfections, charge him an additional fee for each "new case" and keep him in ignorance of his real condition. Whether through ignorance, carelessness or mercenary motives, the disease is allowed to go unrecognized—at least by the victim—the results are, nevertheless, far-reaching and disastrous.

By chronic gonorrhœa, we mean that condition following an acute attack, where at one or more points in the urethra there remain localized foci of inflammation, ready upon the slightest provocation to rekindle the inflammation throughout the urethra. These foci may be found in the anterior urethra where they may be readily relieved, but most frequently, they are found in the deep urethra, from whence the disease is dislodged with the greatest difficulty, not infrequently hanging on for years. Cases are not at all exceptional who present themselves with a history of more or less continuous blenorrhœas, extending over a series of years, the degree varying from a copious discharge to a simple gluing together of the meatus in the morning. Even after the gluing of the meatus has ceased, there may still remain a localized posterior urethritis, capable of transmitting the disease by sexual contact to a healthy mucous membrane.

On examining the urine of such a patient in two or three separate vessels, the first half ounce will contain shreds of pus if the inflammation be confined to the anterior urethra, the second half ounce will contain shreds if posterior urethra is the seat of the inflammation, while the entire quantity will be cloudy if there be coexisting cystitis. Now, this is the test which I usually apply in my office. I have on hand a few glasses in which I have patients pass their urine. By this examination of the urine you can arrive at no definite idea, but a conclusion sufficiently definite to enable you to treat the disease satisfactorily. Where greater accuracy is desirable, the anterior urethra should be first thoroughly irrigated. Then, if the first urine voided contains shreds and flocculi of mucus and pus we know the source is posterior to the cut-off muscle. There may be cases where very slight pressure will force fluid beyond the cut-off muscle, but those are exceptional cases. Microscopical examination will reveal pus corpuscles in abundance. Not only are pus corpuscles found in abundance but also gonococci within the pus cells. On straining they will be found in great abundance.

Now, as to the treatment. During Dr. Nicolson's administration, three or four years ago, we had a discussion on this same subject. I then spoke of deep urethral injections which I had not used up to that time. Keyes had been advocating the deep urethral injections for some time. Ultzman is the man who invented the deep urethral syringe and this is Keyes' modification. The plan of treatment which was advocated is as follows: After the urine had been examined, then, by examination with a bulbous bougie or endoscope, the point was located where the inflammation existed. After the point was definitely located, then with this syringe a strong injection was made, usually one or two minims, the strength varying from one grain of nitrate of silver to the drachm of distilled water, to twenty grains, varying the strength and increasing it as the patient could bear it. Other substances have been used, but nitrate of silver has been the main reliance in this condition, and Keyes and others have advocated the strong injection. Latterly Keyes has advocated an injection, still using this syringe, of a much milder strength, using a larger quantity. He fills the syringe and makes the injection proportionately weaker and injects that behind the cut-off muscle. Then, on washing out the posterior urethra, a certain portion will pass into the bladder. I have used this method and it is a great improvement over the old plan of anterior injections and not a few cases may be relieved by this instrument. The objection I have found is the irritation which it sometimes produces. Here is an instrument which I now use in a large proportion of the cases I treat. This idea is not original with me. I have tried to find the source from whence I got it, but it has been destroyed, or the journal in which it was shown has been lost, and I am not sure who the author of this plan is. It does not carry it out in exactly the same manner, but the idea is the same. I use it because I get so much better results with it than with the other syringe. It is a soft, red rubber catheter velvet eye. The plan of using this instrument is, first to fill the syringe with a solution of nitrate of silver. I keep on hand a stock solution, about 1-1000. For the first injection I dilute this stock solution to about 1-6000 and increase it as the urethra seems to tolerate it, up to the strength desired. I have never used stronger than 1-3000. The patient always feels better afterwards. I have not injected a single case who did not tell me he felt better and would come back and want another injection. The catheter is introduced beyond the cut-off muscle and then the entire quantity of fluid is injected into the posterior urethra. It forces its way back into the bladder, thoroughly distending the posterior urethra and washing the entire bladder. Then the instrument is gradually withdrawn and the fluid injected again. You can do it with one hand. This washes out the whole urethra.

Now, this is the plan of treatment I adopt in these cases. First, if there be an existing anterior urethritis or stricture, that must first be relieved. Then I rely upon the plan of treatment above described.

Now, to state it briefly, I have, within the last few months, treated a number of cases, one gentleman coming from a neighboring town, about fifty-four miles away. He had had gonorrhœa for over six years. He was a nervous wreck. Dr. Hubbard and the physician who came with him assisted me in the operation. As I say, he was a nervous wreck. He believed his kidneys were affected. He was undone morally and physically. He had been repeatedly "cured" of gonorrhœa. He had been in the hands of a dozen or more physicians, and they were as good practitioners as you can find almost anywhere and he had been treated for repeated attacks of gonorrhœa. He finally realized that these were not new attacks and that he had a chronic inflammation there which set up the acute attack on every excess—whether venereal or alcoholic. That gentleman not only had chronic gonorrhœa but he had stricture also. After relieving the stricture, this gonorrhœa persisted and every time he would leave off his injections and leave off the oil of sandalwood he was taking, he would have a discharge on the slightest excess. If he sat up late at night, lost sleep, if he drank anything—whether he indulged in venery or not, the discharge would come back. I did not treat this patient. I gave him one injection. Then he passed from my hands into the hands of the physician who came with him and he carried out the treatment. I had a letter from him a short while after he left, stating that there had not been the slightest discharge. None of those shreds were found in the urine after the second injection. I gave him one injection and his physician carried out the injections afterwards. He used it for three or four weeks and said not a single shred was found in the urine. I mean by shred, not little mucous shreds which are found in normal urine, but those pus flocculi—those thick, heavy shreds which we find in the urine of a patient suffering from this disease. He has since married but has not had any trouble.

I could report a number of other instances, equally as striking as that. Some of them live here and some of them have come from a distance. I had a young man from near Meridian, Miss., who had suffered for four or five years. He had a very slight stricture—in fact, it hardly amounted to any stricture at all. It was relieved by passing in a sound a few times but the discharge persisted. The meatus would be glued up every morning and he told me that for three or four years, every time he had intercourse with a woman, it did not make any difference how pure the woman was, the next morning he would wake up with a blooming urethral discharge.

Those are cases which we meet with every day. Every man who does any genito-urinary practice at all—and every physician does some—will find those cases come right along in his practice. Now, what is the danger of this condition? Not only danger to the patient himself—that is not where the great moral responsibility rests both of the physician and the patient—but if we do not recognize this condition and allow this man to cohabit with a pure innocent woman, he is almost certain to communicate the disease to the woman.

Now, of the horrors of gonorrhœa in women and its ulterior bad effects, and perhaps death it produces, I will not speak. Numbers of cases of pyosalpinx are claimed by our gynecologists to be due to gonorrhœa.

Now, that, in brief, is what I wish to say on this subject. The point I wish to bring up especially is the responsibility of physicians. They are all cases which can be recognized. There is no reason why he should not recognize these cases, and there is no reason why he should not relieve a large proportion of them. I have found several cases I could not cure but as yet I have not found a single patient who has not been benefited by the deep urethral injections. I mean those cases which have lasted for weeks, months or years. There are some, I believe, that will never get well. I remember last year while at the New York Polyclinic, a gentleman told me that the man who lectures there on venereal diseases related a case of chronic gonorrhœa which he had which ran on for more than a year and he said: "The man took sick with typhoid fever, and when he got well his gonorrhœa got well, but that is the only thing I know of that will cure it, and I do not know whether that will cure another case or not." So that is this able physician's opinion about it. Any contribution to the treatment of this disease I think is a valuable addition to the history of medicine, but my results with this plan of treatment have been so remarkable that I wanted to bring it before this society for discussion this evening. (For the discussion see page 214.)

